# Differences in Body Balance According to Body Mass Classification among Brazilian Jiu-Jitsu Athletes

**DOI:** 10.3390/ijerph192316116

**Published:** 2022-12-02

**Authors:** Justyna Leszczak, Ewelina Czenczek-Lewandowska, Justyna Wyszyńska, Justyna Podgórska-Bednarz, Aneta Weres, Rafał Baran, Marta Niewczas, Teresa Pop, Joanna Baran

**Affiliations:** 1 Institute of Health Sciences, Medical College of University of Rzeszow, ul. Kopisto 2a, 35-959 Rzeszów, Poland; 2 Institute of Physical Culture Sciences, Medical College of University of Rzeszow, ul. Kopisto 2a, 35-959 Rzeszów, Poland

**Keywords:** body mass index, jiu-jitsu, martial arts, postural balance, body balance

## Abstract

Background: Body weight is an important aspect in the development of components of physical fitness that can affect athletic performance. The purpose of this study was to examine the differences in body balance of Brazilian Jiu-Jitsu (BJJ) athletes according to body mass classification: underweight/normal weight (UW/NW); overweight/obese (OW/OB). Material and Methods: The study was conducted among 69 BJJ athletes (age 23.26 ± 3.53) and 93 non-practicing adults (age 21.73 ± 2.32). This study was based on a quantitative assessment of body balance on the Zebris PDM platform during two tests, i.e., with eyes open and closed. Results: The total path of the center of pressure (COP TTL) was significantly higher in participants with UW/NW compared to those with OW/OB, both in participants from the study group and control group (*p* < 0.001). COP TTL was significantly lower in UW/NW BJJ athletes than in participants in the control group with the same BMI category (987.4 mm vs. 1238.5 mm and 1080.59 mm vs. 1280.70 mm, respectively) (*p* < 0.001). Conclusions. BJJ training is associated with a better balance in terms of COP TTL in the case of people with normal or underweight. The presence of excess body weight has a positive effect on lower COP TTL values in both practicing and non-practicing people.

## 1. Introduction

The ability to maintain the correct position of the body and balance during fast movement is an important feature of a physically fit person. This skill is particularly important in athletes in whom incorrect or uncontrolled movement may reduce performance or be a cause of injury [1,2]. Balance and the subjective feeling of correct orientation in space depend primarily on the proper integration of vestibular, visual, and proprioceptive stimuli. The stimuli received are processed by the central nervous system and then transferred to the musculoskeletal system, limiting the sway of the body posture in such a way that the projection of the center of mass of the body (COM) is located on the surface of the base of support (BOS) [3]. Balance reactions are compensatory fluctuations in muscle tone that maintain or restore balance in a given position of the body. Primary balance reactions begin to take shape at around 6–8 months of age and develop fully between 18 and 24 months of age. Balance reactions are active for a lifetime with a downward trend after the age of 50 [4,5,6,7]. With time, the efficiency of the components of the balance system deteriorates, but regular training can greatly delay this process [8].

Balance disorders are not always related to the functioning of elements of the balance system, and apart from individual genetic conditions, these processes may be indirectly affected by psychological or environmental conditions. It is necessary to mention the following variables: age, fitness, intake of psychoactive substances or other drugs, spatial orientation disorders, labyrinth diseases, e.g., motion sickness, labyrinthitis, and vestibular neuritis in the balance system [9,10,11,12,13,14].

Body weight is conditioned by both genetic and modifiable environmental factors that are directly transformed into the functional condition of the musculoskeletal system [15]. However, it should be remembered that, along with a range of health problems associated with increased body mass (BM), the increasing prevalence of related comorbidities, including metabolic (e.g., diabetes mellitus, hyperlipidemia, hypertension) and nonmetabolic disorders (e.g., cancer, stroke, depression, polycystic ovary syndrome, fatty liver disease, glomerulopathy, bone fragility, etc.), adiposity and reducing of fitness level are important limiting factors for the realization of regular physical exercise and quality of life [16,17]. At the same time, changes that occur mainly in the musculo-osteoarticular nervous system seem to be important for the entire balance control process. It should be assumed that the appearance of disturbances in the functioning of these systems, together with body weight, can translate into the ability to maintain balance [18].

The correct and adequately fast balance reactions of the body during training or sport competitions should be one of the main training goals of competitors practicing most sports. However, it is particularly important in disciplines that require quick and changeable motions during which balance is often lost, resulting in poor athletic performance. This group includes, among others, athletes who practice martial arts such as judo, taekwondo, karate, Krav Maga, or Brazilian Jiu-Jitsu (BJJ) [19].

In studies carrying out biomechanical evaluation of the relationship between postural control and body mass index among young adults, it was shown that BMI has an impact on postural control during both bipedal posture and one-legged stance. Postural control was found to be negatively correlated with increased adiposity, as the obese BMI group performed significantly worse than the underweight, normal weight, and overweight groups during both tests [18]. Eghbal et al., among Greco-Roman wrestlers, also showed a negative relationship between body mass index (BMI) and body fat content (BFP) on the performance of the players [20]. Body weight and BFP are major performance variables in many sports. Additional body composition measurements are very important for health reasons. However, there are many methods of their assessment [21]. Recent studies show that BMI disorders can affect postural stability. Short height and low body weight affect the position of the center of gravity, which fundamentally affects balance. The results of a chi-square analysis showed a relationship between BMI and static and dynamic balance among students of the Malikussaleh University Martial Arts Club [22].

The current evidence on the relationship of body composition and balance has so far been limited to studies regarding different types of martial arts. To our knowledge, there are no reports on these relationships among BJJ trainees. Therefore, we explored the association between body mass classification and balance among Jiu-Jitsu practitioners. The results of this analysis among BJJ competitors may affect future training selections in study groups.

The aim of this study was to evaluate the relationship between body mass classification and balance in BJJ athletes.

## 2. Materials and Methods

### 2.1. Sample Size

Based on data published by the Central Statistical Office (Poland), there were approximately 836 athletes practicing BJJ between 2017 and 2018 in Podkarpacie, Poland. Assuming a confidence level of 90% and a 5% maximum of error, the required sample size should include at least 63 participants. The initial number of people who were invited to participate in the study was 230 adults (107 amateur BJJ athletes and 123 adults who did not declare BJJ training). A total of 47 subjects did not agree to participate in the study, 16 subjects attended training but experienced injuries, and 5 subjects declared the presence of a chronic disease. Finally, the study group consisted of 69 athletes who practiced BJJ and 93 adults who did not practice, serving as the control group.

### 2.2. Participants

The inclusion criteria in the study group were: 18–34 years of age, written informed consent for examination, regular BJJ training for a minimum of one year, only amateurs trained in BJJ, without diagnosed chronic labyrinth diseases or diseases of the nervous system. The inclusion criteria for the control group were as follows: 18–34 years of age, written informed consent for examination, not practicing professional or amateur sports, no diagnosed chronic labyrinth diseases or diseases of the nervous system. The tests were carried out in athletic clothing without shoes at least 2 hours after a meal.

The exclusion criteria were the presence of metal or electronic implants, menstruation in women, current injury, recent musculoskeletal trauma, epilepsy, labyrinth or nervous system disease, chronic drug use, no informed consent, practicing BJJ training for less than a year prior to participation in the study, practicing another sport or martial art other than BJJ, or practicing BJJ professionally. The exclusion criteria for the control group also included amateur BJJ practice.

### 2.3. Measurements

The study of balance and symmetry of lower limb loading was performed by means of the static posturography method using the Zebris PDM stabilometric platform combined with a computer system (Zebris Medical GmbH: WinSpine 2.3 software). The displacements of the center of gravity were recorded at a frequency of 100 Hz. The method assesses postural sway while a patient is standing in a fixed standing position (static test) based on graphical records of displacements of the point of application of the resultant forces of pressure with feet on the ground (COP—center of pressure) [23,24,25].

The test was carried out in a freestanding position with the upper limbs placed along the trunk with the feet slightly apart at an angle of 30 degrees. In the task with eyes open, the subject focused on a designated point at eye level. The first attempt was carried out under the control of sight, and the second without visual control (eyes closed). Each trial lasted 30 seconds and the recording 20 seconds (the initial 10 seconds were discarded as preparation time to eliminate uncontrolled foot pressure movements) [26]. The COP (center of pressure) sway was recorded in the horizontal plane (horizontal deviation) (COP HD (mm)) and vertical deviation (COP VD [mm]), as well as the total length of the path overcome by the center of foot pressure on the stabilometric platform (COP TTL (mm)). Horizontal deviation (HD) is the average lateral deviation of the feet’s COP from point 0, which is a geometric center of gravity calculated for a given patient (mm). Vertical deviation (VD) is the average anteroposterior deviation of the feet’s COP from point 0 (mm). The markings used correspond to COP HD-ML, the mediolateral component of COP, and COP VD-AP, the anterior-posterior component of COP.

All measurements were made by the same members of the research team in the afternoon before training or competition. The results of the study were recorded in the form of a report on a spreadsheet. The report contained numerical data, as well as graphical images of the loading of the subject’s lower extremities.

Body height was measured with an accuracy of 0.1 cm and body weight with an accuracy of 0.1 kg using a set of electronic medical scales with a RADWAG WPT 60/150 OW height meter. The measurements were carried out under standard conditions where the subject was dressed in athletic clothes without shoes taking a relaxed standing position with the upper limbs placed along the trunk, calcaneal tuberosity, hip joints, shoulder joints, and occipital protuberance of the skull touching the stadiometer, with eyes facing forward. After obtaining the results, body mass index (BMI) was calculated according to the WHO obesity classification, that is, 18.49 kg/m^2^ underweight, 18.5–24.9 kg/m^2^ normal body weight, 25–29.9 kg/m^2^ overweight, and 30 kg/m^2^ or over obese.

### 2.4. Statistical Analysis

The results are expressed in the form of arithmetic means, standard deviations (SD), median values, quartiles, interquartile ranges, and 95% confidence interval (CI). Comparison between the control group and the test group and the existence of differences between age groups or weight categories was made using the *t*-Student for independent samples, the independent variance estimation test (Welch), or the Mann–Whitney U test (Statistica 13.3, TIBCO, Palo Alto, CA, USA). The occurrence of correlations between variables and balance parameters in the study and control groups was calculated using Spearman’s rank correlation or Pearson’s correlation. The selection of tests was made on the basis of the normality of distribution of variables (verified by the Shapiro–Wilk test). Differences in *p* < 0.05 were considered statistically significant.

## 3. Results

The general characteristics of the groups are presented in Table 1. The average age of the study and control group participants was 23.26 ± 3.53 and 21.73 ± 2.32, respectively. The average BMI of the participants in the study group was 23.68 kg/m^2^ and the BFP was 10.59%, while the BMI of the participants in the control group was lower (21.40 kg/m^2^) and the BFP higher (19.30%).

Table 2 shows the differences in the values of the COP TTL, COP HD, and COP VD depending on the age of the participants in the study and control groups. During the analysis, we compared differences between two ages groups (18–24 years old vs. 25–34 years old). The total value is only for information. The median HD value of COP during open eye tests was significantly higher in younger participants (18–24 years) compared to older participants (25–34 years) in both the study and control groups. During the test with eyes closed, the median value of COP HD was significantly higher in the younger participants compared to the older ones in the study group, and the opposite in the control group—the median value of COP HD was higher in the older participants compared to the younger.

Table 3 presents the differences in the COP TTL, COP HD, and COP VD values between the study and the control group, considering the age of the participants. The median COP TTL during both open and closed eyes tests was significantly lower in participants in the study group compared to the control group (957.60 mm vs. 1152.40 mm, and 939.10 mm vs. 1226.80 mm, respectively). Similar results were obtained when analyzing participants according to age, but statistically significant differences were observed only for the younger group (18–24 years).

The median COP HD and COP VD during the tests with open and closed eyes were significantly higher in participants from the study group compared to the control group. When analyzing these differences considering age, similar results were obtained for younger participants compared to older ones.

During the analysis, we compared differences between two body mass categories (UW/NW vs. OW/OB). The total value is only for information. The differences in the values of COP TTL, COP HD, and COP VD between the study group participants and the control group, depending on the weight category, are presented in Table 4. The median COP TTL during open and closed eyes tests was significantly higher in participants with UN/NW weight compared to those with OW/OB, both in participants in the study group and in the control group.

Table 5 presents the differences in the values of COP TTL, COP HD, and COP VD between the study and the control group, considering the category of body weight of the participants. The median COP TTL during both open and closed eyes tests was significantly lower in underweight/normal participants of the study group compared to underweight/normal weight participants of the control group (987.4mm vs. 1238.5 mm and 1080.59 mm vs. 1280.70 mm, respectively). The median HD COP during the tests with open and closed eyes and COP VD during the tests with closed eyes were significantly higher in underweight/normal participants in the study group compared to the control group.

Table 6 shows the relationship between COP TTL, COP HD, and COP VD and selected factors in the study and control groups. In the test with eyes open, negative correlations were observed between the age of the participants with COP HD and COP VD in the study group. In the control group, negative correlations were observed between the age of the participants with COP TTL and COP HD. Furthermore, negative correlations were observed between BFP and COP TTL, both in the study and in the control groups.

In the test with eyes closed, negative correlations were observed between the age of the participants with COP HD and COP VD in the study group. In the control group, a negative correlation was observed between the age of the participants and the COP VD. Furthermore, negative correlations were observed between BFP and COP TTL in both the study and control groups.

## 4. Discussion

BJJ is a martial art that requires strong and flexible muscles, as well as good balance capabilities [27]. For this group of competitors, maintenance of correct balance of the body, both during training and, above all, in competition, is a component of successful performance. It is one of the factors that can determine the effectiveness of techniques in attack and defense presented by the competitor [28]. The optimal criteria for an effective BJJ technique are based on the full use of biomechanical rules, which mainly involve the balance system. During each maneuver, the competitor must maintain a stable body posture that will not allow him to lose or disturb his balance [29].

Many researchers confirm that long-term training based on exercises that disturb balance has a positive effect on shaping postural control [30,31]. This is also confirmed by our own research where BJJ practitioners presented significantly better balance (refers to COP TTL) capabilities compared to non-practicing individuals within the scope of both tests, with eyes closed and open. As expected, the results of the trial with closed eyes were lower due to the fact that in the case of excluding the visual system, there was a greater involvement of other sensory systems, that is, the sensory-motor and vestibular system, which was associated with the need for a larger correction of body balance. Similarly, other authors indicate a much higher level, especially in dynamic balance, among athletes practicing various martial arts compared to control groups [32]. Truszczyska et al. describe the positive impact of karate training on motor development and postural control of school-age children, their increased mediolateral stability, and lower COP values [33]. However, in our study, the reverse was found, as people practicing BJJ had higher values of COP HD and COP VD in relation to non-practicing people.

Analyzing various factors that may have an adverse effect on the balance of martial arts athletes, the most common include dysfunctions of the sense organs, for example, weak sight or a hearing deficiency [34]. Bednarczuk et al. suggested reduced static balance in athletes with eye disorders practicing various sports, especially in those who trained less than 5 hours a week. The type of sport practiced, the degree of dysfunction, and the athletic experience did not significantly distinguish the studied groups; the quality of sport training had a greater impact on balance capabilities [35]. It is worth mentioning that no unambiguous answers have been found concerning the impact of body mass index on balance capabilities so far. Interestingly, it was noticed that older people practicing BJJ had better balance (smaller values of COP VD and HD) than younger practitioners in the tests with both eyes open and closed. We cannot associate this with training because no correlation with training experience was found. Furthermore, people with a higher body fat content had lower values of the COP TTL, which in turn is associated with subsequent results, dividing the group according to the body mass category.

Previous studies of balance have been conducted most often in older, obese individuals with neurological diseases or athletes of various disciplines. For example, the somatic characteristics, aerobic and anaerobic profiles, handgrip strength, muscle power, flexibility, or reaction time of BJJ competitors are well known, but little is known about balance capabilities [36]. In our study, it was found that people with increased body weight had a shorter COP TTL in both study and control groups in both trials. On the other hand, significantly higher COP TTL values were found in non-practicing subjects with normal or reduced body mass. In terms of tilts to the sides and in the front-back direction, the situation is opposite, so we cannot clearly determine which group was characterized by better balance.

Regular BJJ training helps maintain a healthy body mass composition [36]. In our study, BJJ competitors had almost fifty percent less body fat content compared to adults who do not practice sports. Similar conclusions in relation to adults training in karate were presented by I. Bertini et al. indicating that cyclic karate training allows athletes to maintain the appropriate level of body mass index for the athlete’s category [37]. Moreover, Barley insists that maintaining a proper body weight to qualify for a particular weight category in sport is particularly important for competitors practicing martial arts such as BJJ [38].

Greve et al. reported that a 20% increase in BMI is positively correlated with an increase in postural instability [39]. Moreover, Nascimento notes that young people with obesity are characterized by a much worse balance reaction, and they need more time to perform given activities and are more likely to fall than people with normal weight. However, the largest differences between the two groups were observed only in terms of dynamic balance [40]. Similarly, Gao et al. demonstrate a strong association of reduced dynamic balance with an increase in BMI, but the study group was composed of elderly individuals whose balance capacities may already have decreased and the condition of excess weight and obesity can deepen and accelerate this process [41]. These results are opposite to our results. This leads to the continuation of the research, especially in a larger group of subjects, taking into account the proportion of fat and muscle tissue in relation to balance. BMI itself may not be an entirely appropriate factor, especially in athletes with developed muscle mass [42].

It is worth mentioning that the issue of obesity and balance reactions was also raised by Benetti et al. The authors made an interesting attempt to examine the balance and flexibility of obese patients after bariatric surgeries, but in their opinion a significant reduction in body weight of the participants did not have an effect on static balance of the subjects. However, this situation may be explained by the lack of rapid adaptation of the proprioceptive system after surgery [43].

Furthermore, a detailed analysis that presented individual components of body mass and balance reactions in young people was also performed by Alonso et al. Body weight and BMI did not have a significant impact on maintaining balance during the tests with closed and open eyes. The authors point to other anthropometric characteristics that may be more related to balance than body weight, e.g., body height and bone mineral composition [44].

Brazilian Jiu-Jitsu athletes performed better in strength tests (static strength, relative strength, shoulder girdle strength, functional strength). High correlations between training load and physical fitness level were found in flexibility and strength tests in BJJ athletes and most strength tests in Muay Thai athletes [45]. Thus, strength and conditioning programs that target specific components, rather than mimic the characteristics of the sport, are often preferable [46]. These results may explain why no clear direction was obtained in the balance analysis in BJJ competitors. The specificity of the training means that mainly strength is developed, rather than balance.

Considered within the context of training, in addition to the need to constantly control body weight in BJJ, it is important to shape high balance capabilities and postural control skills through the steady introduction of balance exercises [47]. In addition to the traditional instruments used in these exercises, i.e., balance platforms or gymnastic balls, training may employ newer technologies that aid in developing balance reactions. For example, Dunsky et al. describe the use of the ‘FIFA 11 +’ program, i.e., a complete warm-up package that combines cardiovascular activation and preventive neuromuscular exercises in the training of young football players, which significantly improved the static balance of the athletes studied [48].

High performance in BJJ is a derivative of the comprehensive development of all motor skills of the competitor, and balance reactions constitute an important component. BJJ training helps to maintain normal body weight, but it should be constantly monitored because irregularities in this area may indirectly affect reduction or improvement of a competitor’s balance functions, which is important for performance.

## 5. Conclusions

The research carried out allows us to conclude that BJJ training is associated with better balance in terms of COP TTL in the case of people with normal or underweight. At the same time, these people show greater deflections of the COP in the horizontal and vertical planes. In addition, the presence of excess body weight has a positive effect on lower COP TTL values in both practicing and non-practicing people. In summary, these conclusions prompt further research to enable us to clearly determine the impact of BJJ training and body weight category on balance.

## Figures and Tables

**Table 1:** Characteristics of the groups—descriptive statistics.

Variables							
	$\bar{x}$	SD	Me	Q1	Q3	IQR	95% CI
**Study group (n = 69)**							
Age (years)	23.26	3.53	23.00	20.00	26.00	6.00	22.41; 24.11
Body mass (kg)	75.22	11.04	74.80	67.00	82.80	15.80	72.57; 77.88
Height (cm)	175.52	22.06	180.00	175.00	183.00	8.00	170.22; 180.82
BMI (kg/m^2^)	23.68	2.75	23.40	21.80	25.50	3.70	23.02; 24.34
BFP	10.59	5.06	9.60	7.20	12.90	5.70	9.38; 11.81
Training period (months)	40.99	51.54	24.00	12.00	36.00	24.00	28.60; 53.37
**Control group (n = 93)**							
Age (years)	21.73	2.32	21.00	20.00	22.00	2.00	21.25; 22.21
Body mass (kg)	60.81	13.25	57.60	51.90	67.70	15.80	58.08; 63.54
Height (cm)	168.70	7.81	168.00	164.00	173.00	9.00	167.09; 170.31
BMI (kg/m^2^)	21.40	3.45	20.60	19.20	23.00	3.80	20.69; 22.11
BFP	19.30	7.11	18.40	13.80	25.00	11.20	17.84; 20.77
Training period (months)	n.a.	n.a.	n.a.	n.a.	n.a.	n.a.	n.a.

BFP, body fat percentage; BMI, body mass index; $\bar{x}$, arithmetic average; Me, median; Q1, lower quartile; Q3, upper quartile; SD, standard deviation; IQR, interquartile range; CI, confidence interval.

**Table 2:** Differences in the values of the center-of-pressure depending on the age of the participants.

**Study Group**									
	**COP TTL (mm)**			**COP HD (mm)**			**COP VD (mm)**		
	**Open Eyes**								
	**n**	**Me**	**IQR**	**n**	**Me**	**IQR**	**n**	**Me**	**IQR**
Total	69	957.60	289.50	69	2.80	1.40	69	4.20	3.30
18–24 years	44	949.45	269.10	44	2.95	1.75	44	4.45	3.85
25–34 years	25	957.60	314.70	25	2.30	1.40	25	3.80	2.30
*p*	U = 510.50 *p* = 0.622 ***			U = 323.50 *p* = 0.005 ***			U = 455.00 *p* = 0.236 ***		
	**Closed Eyes**								
	**n**	**Me**	**IQR**	**n**	**Me**	**IQR**	**n**	**Me**	**IQR**
Total	69	939.10	298.00	69	2.40	1.20	69	4.70	2.50
18–24 years	44	960.95	272.65	44	2.50	1.45	44	5.10	2.10
25–34 years	25	932.70	376.20	25	2.20	0.80	25	4.50	2.20
*p*	U = 495.00 *p* = 0.492 ***			U = 370.00 *p* = 0.025 ***			U = 394.00 *p* = 0.051 ***		
**Control Group**									
	**COP TTL (mm)**			**COP HD (mm)**			**COP VD (mm)**		
	**Open eyes**								
	**n**	**Me**	**IQR**	**n**	**Me**	**IQR**	**n**	**Me**	**IQR**
Total	93	1152.40	457.10	93	2.40	1.10	93	3.80	1.80
18–24 years	82	1194.20	451.00	82	2.40	1.40	82	3.70	1.80
25–34 years	11	1074.30	199.0	11	1.90	1.10	11	4.40	2.70
*p*	U = 351.00 *p* = 0.237 ***			U = 283.00 *p* = 0.046 ***			U = 388.00 *p* = 0.457 ***		
	**Closed Eyes**								
	**n**	** x **	**SD**	**n**	**Me**	**IQR**	**n**	**Me**	**IQR**
Total	93	1227.06	289.21	93	2.00	1.20	93	4.00	1.60
18–24 years	82	1234.45	284.77	82	2.00	1.30	82	3.85	1.80
25–34 years	11	1171.95	329.97	11	2.30	0.80	11	4.40	1.70
*p*	T = 0.686 *p* = 0.495 *			U = 404.00 *p* = 0.580 ***			U = 340.00 *p* = 0.189 ***		

* *t*-Student for independent samples; *** Mann–Whitney U test, significant associations are highlighted in bold; n, sample size.

**Table 3:** Differences in the values of the center-of-pressure considering the age of the participants.

	Study Group			Control Group			*p*
	n	Me	IQR	n	Me	IQR	
**COP TTL (mm)—Open Eyes**							
Total	69	957.60	289.50	93	1152.40	457.10	U = 1865.00 *p* < 0.001 ***
18–24 years	44	949.45	269.10	82	1194.20	451.00	U = 1039.00 ***p* < 0.001** ***
25–34 years	25	957.60	314.70	11	1074.30	199.0	U = 101.00 *p* = 0.216 ***
**COP TTL (mm)—Closed Eyes**							
	**n**	**Me**	**IQR**	**n**	**Me**	**IQR**	
Total	69	939.10	298.00	93	1226.80	481.60	U = 1771.00 *p* < 0.001 ***
18–24 years	44	960.95	272.65	82	1252.95	481.60	U = 1003.00 *p* < 0.001 ***
25–34 years	25	932.70	376.20	11	1131.60	613.60	U = 93.00 *p* = 0.131 ***
**COP HD (mm)—Open Eyes**							
	**n**	**Me**	**IQR**	**n**	**Me**	**IQR**	
Total	69	2.80	1.40	93	2.40	1.10	U = 2497.00 *p* = 0.016 ***
18–24 years	44	2.95	1.75	82	2.40	1.40	U = 1204.50 *p* < 0.001 ***
25–34 years	25	2.30	1.40	11	1.90	1.10	U = 89.50 *p* = 0.103 ***
**COP HD (mm)—Closed Eyes**							
	**n**	**Me**	**IQR**	**n**	**Me**	**IQR**	
Total	69	2.40	1.20	93	2.00	1.20	U = 2314.50 *p* = 0.003 ***
18–24 years	44	2.50	1.45	82	2.00	1.30	U = 1093.50 *p* < 0.001 ***
25–34 years	25	2.20	0.80	11	2.30	0.80	U = 136.50 *p* = 0.986 ***
**COP VD (mm)—Open Eyes**							
	**n**	**Me**	**IQR**	**n**	**Me**	**IQR**	
Total	69	4.20	3.30	93	3.80	1.80	U = 2602.00 *p* = 0.040 ***
18–24 years	44	4.45	3.85	82	3.70	1.80	U = 1323.50 *p* = 0.014 ***
25–34 years	25	3.80	2.30	11	4.40	2.70	U = 132.50 *p* = 0.877 ***
**COP VD (mm)—Closed Eyes**							
	**n**	**Me**	**IQR**	**n**	**Me**	**IQR**	
Total	69	4.70	2.50	93	4.00	1.60	U = 2055.00 *p* < 0.001 ***
18–24 years	44	5.10	2.10	82	3.85	1.80	U = 964.50 *p* < 0.001 ***
25–34 years	25	4.50	2.20	11	4.40	1.70	U = 129.00 *p* = 0.784 ***

*** Mann–Whitney U test, significant associations are highlighted in bold.

**Table 4:** Differences in the values of the center-of-pressure depending on the weight category.

	Study Group								
	COP TTL (mm)			COP HD (mm)			COP VD (mm)		
	**Open Eyes**								
	**n**	**Me**	**IQR**	**n**	**Me**	**IQR**	**n**	**Me**	**IQR**
UW/NW	49	987.40	307.70	49	2.80	1.40	49	4.00	3.60
OW/OB	20	786.55	199.55	20	2.80	1.65	20	4.50	3.00
Total	69	957.60	289.50	69	2.80	1.40	69	4.20	3.30
*p*	U = 182.50 *p* < 0.001 ***			U = 480.50 *p* = 0.905 ***			U = 482.00 *p* = 0.921 ***		
	**Closed Eyes**								
	**n**	$\bar{x}$	**SD**	**n**	**Me**	**IQR**	**n**	$\bar{x}$	**SD**
UW/NW	49	1080.59	234.94	49	2.40	1.20	49	4.94	1.54
OW/OB	20	834.06	102.53	20	2.25	1.20	20	5.05	1.61
Total	69	1009.13	233.66	69	2.40	1.20	69	4.97	1.55
*p*	t = 6.065 *p* < 0.001 **			U = 463.50 *p* = 0.731 ***			t = −0.261 *p* = 0.795 *		
**Control Group**									
	**COP TTL (mm)**			**COP HD (mm)**			**COP VD (mm)**		
**Open eyes**									
	**n**	**Me**	**IQR**	**n**	**Me**	**IQR**	**n**	**Me**	**IQR**
UW/NW	80	1238.50	376.40	80	2.40	1.25	80	3.70	1.85
OW/OB	13	907.10	278.70	13	2.20	1.30	13	4.30	2.30
Total	93	1152.40	457.10	93	2.40	1.10	93	3.80	1.80
*p*	U = 157.50 *p* < 0.001 ***			U = 452.50 *p* = 0.458 ***			U = 434.00 *p* = 0.343 ***		
**Closed Eyes**									
	**n**	$\bar{x}$	**SD**	**n**	**Me**	**IQR**	**n**	**Me**	**IQR**
UW/NW	80	1280.70	269.86	80	2.00	1.20	80	3.85	1.65
OW/OB	13	896.99	156.91	13	2.10	1.20	13	5.00	1.60
Total	93	1227.06	289.21	93	2.00	1.20	93	4.00	1.60
*p*	t = 7.246 *p* < 0.001 *			U = 483.50 *p* = 0.690 ***			U = 328.50 *p* = 0.034 ***		

NW, normal weight; OB, obesity; OW, overweight; UW, underweight. * *t*-Student for independent samples; ** test with independent variance estimation (Welch), *** Mann–Whitney U test, significant associations are highlighted in bold.

**Table 5:** Differences in the values of the center-of-pressure between the study and control groups considering the body weight category.

	**Study Group**			**Control Group**			***p***
**Body Mass Category**	**n**	**Me ^a^** ***x* ^b^**	**IQR ^a^** **SD ^b^**	**n**	**Me ^a^** ***x* ^b^**	**IQR ^a^** **SD ^b^**	
**COP TTL (mm)—Open Eyes**							
UW/NW	49	987.40 ^a^	307.70 ^a^	80	1238.50 ^a^	376.40 ^a^	U = 1197.00 *p* < 0.001 ***
OW/OB	20	807.08 ^b^	112.09 ^b^	13	880.54 ^b^	157.04 ^b^	t = −1.570 *p* = 0.127 *
**COP TTL (mm)—Closed Eyes**							
	**n**	**Me ^a^** ***x* ^b^**	**IQR ^a^** **SD ^b^**	**n**	**Me ^a^** ***x* ^b^**	**IQR ^a^** **SD ^b^**	
UW/NW	49	1080.59 ^b^	234.94 ^b^	80	1280.70 ^b^	269.86 ^b^	t = −4.289 *p* < 0.001 *
OW/OB	20	834.06 ^b^	102.53 ^b^	13	896.99 ^b^	156.91 ^b^	T = −1.398 *p* = 0.172 *
**COP HD (mm)—Open Eyes**							
	**n**	**Me ^a^** ***x* ^b^**	**IQR ^a^** **SD ^b^**	**n**	**Me ^a^** ***x* ^b^**	**IQR ^a^** **SD ^b^**	
UW/NW	49	2.80 ^a^	1.40 ^a^	80	2.40 ^a^	1.25 ^a^	U = 1550.50 *p* = 0.047 ***
OW/OB	20	2.88 ^b^	1.03 ^b^	13	2.27 ^b^	1.01 ^b^	t = 1.666 *p* = 0.106 *
**COP HD (mm)—Closed Eyes**							
	**n**	**Me**	**IQR**	**n**	**Me**	**IQR**	
UW/NW	49	2.40	1.20	80	2.00	1.20	U = 1413.00 *p* = 0.008 ***
OW/OB	20	2.57	1.00	13	2.04	1.00	U = 92.00 *p* = 0.167 ***
**COP VD (mm)—Open Eyes**							
	**n**	**Me**	**IQR**	**n**	**Me**	**IQR**	
UW/NW	49	4.00	3.60	80	3.70	1.85	U = 1564.00 *p* = 0.055 ***
OW/OB	20	4.50	3.00	13	4.30	2.30	U = 120.50 *p* = 0.740 ***
**COP VD (mm)—Closed Eyes**							
	**n**	**Me ^a^** ***x* ^b^**	**IQR ^a^** **SD ^b^**	**n**	**Me ^a^** ***x* ^b^**	**IQR ^a^** **SD ^b^**	
UW/NW	49	4.70 ^a^	2.40 ^a^	80	3.85 ^a^	1.65 ^a^	U = 1184.50 *p* < 0.001 ***
OW/OB	20	5.05 ^b^	1.61 ^b^	13	4.69 ^b^	1.16 ^b^	t = 0.683 *p* = 0.500 *

NW, normal weight. OB, obesity. OW, overweight. UW, underweight. * *t*-Student for independent samples. *** Mann–Whitney U test, significant associations are highlighted in bold.

**Table 6:** Relationship between the values of the center-of-pressure and selected factors in the study and control groups.

	**Study Group**			**Control Group**		
**Variables**	**COP TTL (mm)**	**COP HD (mm)**	**COP VD (mm)**	**COP TTL (mm)**	**COP HD (mm)**	**COP VD (mm)**
**Open Eyes**						
**Age**	−0.08 (*p* = 0.511)	**−0.33** **(*p* = 0.005)**	**−0.25** **(*p* = 0.034)**	**−0.24** **(*p* = 0.018)**	**−0.22** **(*p* = 0.035)**	−0.10 (*p* = 0.359)
**Body height (cm)**	−0.07 (*p* = 0.591)	0.10 (*p* = 0.434)	0.02 (*p* = 0.887)	−0.12 (*p* = 0.266)	0.02 (*p* = 0.829)	0.11 (*p* = 0.275)
**Training period (months)**	−0.05 (*p* = 0.691)	−0.09 (*p* = 0.481)	−0.17 (*p* = 0.152)	-	-	-
**BFP**	**−** **0.41** **(*p* < 0.001)**	−0.01 (*p* = 0.943)	0.06 (*p* = 0.647)	**−0.41** **(*p* < 0.001)**	−0.05 (*p* = 0.639)	0.02 (*p* = 0.876)
**Age**	−0.11 (*p* = 0.371)	**−0.27** **(*p* = 0.026)**	**−0.27** **(*p* = 0.024)**	−0.13 (*p* = 0.225)	−0.01 (*p* = 0.892)	**0.23** **(*p* = 0.027)**
**Body height (cm)**	−0.11 (*p* = 0.375)	−0.11 (*p* = 0.353)	−0.10 (*p* = 0.415)	−0.08 (*p* = 0.452)	0.03 (*p* = 0.750)	0.10 (*p* = 0.365)
**Training period (months)**	−0.01 (*p* = 0.950)	−0.10 (*p* = 0.395)	−0.11 (*p* = 0.349)	-	-	-
**BFP**	**−0.4** **1** **(*p* < 0.001)**	0.02 (*p* = 0.858)	0.01 (*p* = 0.921)	**−0.38** **(*p* < 0.001)**	−0.11 (*p* = 0.276)	−0.10 (*p* = 0.362)

BFP, body fat percentage; in green—Pearson correlation results, other results—Spearman correlation; significant associations are highlighted in bold.

## Data Availability

The data presented in this study are available on request from the corresponding author.
